# Development of an Indirect ELISA Based on a Recombinant Chimeric Protein for the Detection of Antibodies against Bovine Babesiosis

**DOI:** 10.3390/vetsci5010013

**Published:** 2018-01-23

**Authors:** José Manuel Jaramillo Ortiz, Valeria Noely Montenegro, Sofía Ana María de la Fournière, Néstor Fabián Sarmiento, Marisa Diana Farber, Silvina Elizabeth Wilkowsky

**Affiliations:** 1 Instituto de Biotecnología, Centro de Investigaciones en Ciencias Veterinarias y Agronómicas, Instituto Nacional de Tecnología Agropecuaria, De Los Reseros y Dr. Nicolás Repetto s/no., Hurlingham 1686, Buenos Aires, Argentina; Montenegro.valeria@inta.gob.ar (V.N.M.); delafourniere.sofia@inta.gob.ar (S.A.M.d.l.F.); farber.marisa@inta.gob.ar (M.D.F.); wilkowsky.silvina@inta.gob.ar (S.E.W.); 2 Estación Experimental Agropecuaria Mercedes, Instituto Nacional de Tecnología Agropecuaria, Juan Pujol al Este, s/n, Corrientes CP 3470, Argentina; nestor.sarmiento@inta.gob.ar

**Keywords:** bovine babesiosis, multi-epitope recombinant antigen, indirect ELISA, seroprevalence

## Abstract

The current method for *Babesia* spp. serodiagnosis based on a crude merozoite antigen is a complex and time-consuming procedure. An indirect enzyme-linked immunosorbent assay (iELISA) based on a recombinant multi-antigen of *Babesia bovis* (rMABbO) was developed for detection of antibodies in bovines suspected of infection with this parasite. The multi-antigen comprises gene fragments of three previously characterized *B. bovis* antigens: MSA-2c, RAP-1 and the Heat Shock protein 20 that are well-conserved among geographically distant strains. The cutoff value for the new rMABbo-iELISA was determined using 75 known—positive and 300 known—negative bovine sera previously tested for antibodies to *B. bovis* by the gold-standard ELISA which uses a merozoite lysate. A cutoff value of ≥35% was determined in these samples by receiver operator characteristic (ROC) curve analysis, showing a sensitivity of 95.9% and a specificity of 94.3%. The rMABbo-iELISA was further tested in a blind trial using an additional set of 263 field bovine sera from enzootic and tick-free regions of Argentina. Results showed a good agreement with the gold standard test with a Cohen’s kappa value of 0.76. Finally, the prevalence of bovine babesiosis in different tick enzootic regions of Argentina was analyzed where seropositivity values among 68–80% were obtained. A certain level of cross reaction was observed when samples from *B. bigemina* infected cattle were analyzed with the new test, which can be attributed to shared epitopes between 2 of the 3 antigens. This new rMABbo-iELISA could be considered a simpler alternative to detect anti *Babesia* spp. antibodies and appears to be well suited to perform epidemiological surveys at the herd level in regions where ticks are present.

## 1. Introduction

The intraerythrocytic protozoan parasites *Babesia bovis* and *B. bigemina* are the main causative agents of bovine babesiosis. The disease is widespread in tropical and sub-tropical regions of the world where the main tick vector *Rhipicephalus* (*Boophilus*) *microplus* is present [1]. In Argentina, the economical losses in the livestock industry due to treatments and prevention of babesiosis exceed US$38 million per year [2]. All babesial parasites may cause anemia, but *B. bovis* infection causes a severe disease characterized by hemolytic anemia, neurological and kidney damage, respiratory shock and high mortality in adult bovines [3]. A live attenuated vaccine against bovine babesiosis is commercially available in many countries and is currently used. This vaccine can be used safely only in calves under 12 months of age living in enzootically unstable areas [4]. The vaccine is usually administered as a bivalent formula with each *B. bovis* and *B. bigemina* attenuated strains.

In order to attain enzootic stability to *Babesia* spp., at least 75% of the herd should have been bitten at early ages by infected ticks and develop serum antibodies against both *Babesia* species [5]. In this context, the main application of serological tests is to determine the presence of anti-*Babesia* antibodies generated after natural infection of calves or after vaccination. Among the most common tests are the indirect fluorescent antibody test (IFAT) and enzyme-linked immunosorbent assays (ELISA). Competitive ELISA tests were developed for detecting antibodies against both *Babesia* species [6,7] and indirect ELISAs have also been developed using different recombinant antigens [8,9]. All these tests have shown variable results but none of them is available in the market at this time. For this reason, these developments have not replaced the use of an iELISA based on a crude merozoite lysate (MZ-iELISA). This method relies on the maintenance of in vitro *Babesia* cell cultures and purification of merozoites, a complex and time-demanding procedure.

In this work we evaluated the performance of an iELISA using a novel chimerical multi-antigen previously developed in our laboratory [10]. This recombinant multi-antigen (rMABbo) was developed as a vaccine candidate and comprises gene fragments with B and T cell epitopes of three *B. bovis* antigens: MSA-2c, RAP-1 and the Heat Shock protein 20. Unexpectedly, we found that the rMABbo was recognized by antibodies present in bovines naturally infected with different *B. bovis* isolates, suggesting a potential usefulness of this chimeric protein as a serological tool. The aim of this study was to evaluate the performance of the rMABbo in an iELISA format as a simple and rapid alternative to detect *Babesia* antibodies in cattle.

## 2. Materials and Methods

### 2.1. Expression and Purification of the Recombinant Multi-Antigen of B. bovis, rMABbo

In order to obtain the rMABbo, the fragments of *msa-2c*, *rap-1* and *hsp20* (GenBank AY052542.1, AF030062.1, and AF331455.1, respectively) were firstly amplified by PCR and sequentially ligated into a pCR^®^8/GW-TOPO^®^TA Gateway cloning kit vector (Invitrogen Corp, Carlsbad, CA, USA). Then, the single genetic sequence encoding the rMABbo was transferred directionally into pDEST 17™ vector and expressed in *E. coli* strain BL21 AI™ (both from Invitrogen) as previously described [10]. The rMABbo contains a 6X Histidine tag and was purified under denaturing conditions with a Ni-Agarose resin (Probond, Invitrogen) according to the manufacturer’s instructions. Total protein was quantified with a BCA commercial kit (Pierce, Rockford, IL, USA) and stored in aliquots at −20 °C until used.

### 2.2. Origin of Serum Samples, Study Sites and Ethics Statement

For the calculation of the cutoff value, the receiver operator characteristic (ROC) analysis was used with a set of known—positive and known—negative sera. The known—positive sera (*n* = 75) were from *B. bovis* experimentally-infected bovines inoculated with 10^7^ infected erythrocytes of the R1A attenuated strain.

Cattle used for this experimental infection were 10-months-old *Bos taurus* mixed breed, which were tested previously by the MZ-iELISA [11] for the absence of anti *B. bovis* antibodies before use. Forty- five days after inoculation, all serum samples were confirmed positive by the MZ-iELISA and were the samples used in this study. The known—negative serum samples (*n* = 300) used here were from up to 6-month-old Jersey/Holstein crossbred calves born and raised in a dairy farm located in Tandil [12] located in the Buenos Aires province of Argentina. This province is below parallel 30° S, a tick-free area of the country. These sera were also negative for the MZ-iELISA.

For the seroprevalence analysis, only the rMABbo-iELISA was used. Samples were from bovines from the Argentinean provinces of Corrientes (*n* = 89) and Misiones (*n* = 67) which are both above parallel 30° S and enzootic for the *Rhipicephalus microplus* tick. Animals were Angus/Brangus crossbred and had never been vaccinated with the bivalent live vaccine against bovine babesiosis.

An additional set of serum samples was used in a blind test to estimate concordance by the Cohen’s kappa value. For this, a total of 263 sera were evaluated separately by both rMABbo-iELISA and MZ-iELISA as the gold standard. These samples were obtained from bovines from the provinces of Corrientes (*n* = 115) and Misiones (*n* = 67). Both provinces are above parallel 30° S where ticks are enzootic. Eighty-one sera cattle from Tandil region (*n* = 81) were also included to test specificity, a set of *B. bigemina* (*n* = 47) and *Anaplasma marginale* (*n* = 5) serologically positive sera were used. All these sera were tested negative for *B. bovis* antibodies by the current MZ-iELISA.

All the serum samples used in this work were obtained by cattle manually restrained (<5 min) and blood samples were aseptically collected by jugular venipuncture (Vacutainer™, Becton Dickinson, Franklin Lakes, NJ, USA, <0.0005% blood volume; one sampling per animal).

The protocol for animal handling and venipuncture was performed following the guidelines of the Institutional Committee for the Use and Care of Experimentation Animals (protocol approval No. 025/2011). All samples came from privately owned herds and were sampled with the approval of the owners.

### 2.3. Pre-Adsorption of Serum Samples

Cattle sera were incubated at 37 °C for 2 h in pre-adsorption solution to avoid unspecific binding of bovine antibodies [13]. The solution was 5% (*w*/*v*) non-fat dried milk and 100 μg/mL of bacterial culture lysate supernatant in 0.5% (*v*/*v*)-solution of Tween 20 in 1× PBS. Briefly, the bacterial lysate was obtained from 50 mL of overnight *Escherichia coli* culture of strain BL21 AI™ (Invitrogen). Then, the culture was harvested and centrifuged at 17,257 *g* during 10 min at 4 °C. The supernatant was discarded and the pellet resuspended with gentle agitation for 2 h at 4 °C in 4 mL of lysis buffer (100 mM Tris HCl, pH: 7.5; 500 mM NaCl; 20% glycerol; 1% Triton X-100; 20 mM imidazole (pH: 7.4); plus 1 mg/mL Lysozyme and 0.5 mM phenylmethylsulfonyl fluoride). The suspension was sonicated by three cycles (1 min/cycle) and centrifuged at 14,501 *g* during 30 min at 4 °C. The supernatant was separated from the pellet and used. Total protein was quantified with a BCA commercial kit (Pierce, Rockford, IL, USA) and stored at −20 °C until used.

### 2.4. Indirect ELISA Procedure

The MZ-iELISA was performed as described by de Echaide et al., 1995. This technique has been validated by the OIE—World organization for Animal Health as a gold standard method for serological diagnosis. For the rMABbo-iELISA a standard protocol was set up. Different concentrations of antigen (10, 20, 30 and 40 ng) and serum dilutions (1:10, 1:20, 1:50 and 1:100) were previously checked in triplicates in order to optimize the assay conditions (data not shown). Immulon 2 HB Flat Bottom Micro Titer plates (Nunc) were coated overnight at 4 °C with purified rMABbo (20 ng/well) in coating buffer (15 mM Na_2_CO_3_, 35 mM NaHCO_3_, 0.05% Na-azide, (pH: 9.6). The plates were blocked for 1 h at 37 °C with 100 μL of the pre-adsorption solution.

Pre-adsorbed serum samples (1:50 dilution) were added after 3 washes with 0.05% Tween—20 in 1× PBS and the plates were incubated for 1 h at 37 °C. The plates were washed again with 0.05% Tween—20 1× PBS and incubated with horseradish peroxidase-conjugated goat anti-bovine IgG (Sigma-Aldrich, Saint Louis, MI, USA) for 1 h at 37 °C. The reaction was developed by the addition of 100 μL/well of OptEIA™ TMB Substrate Reagent Set (Pharmigen) and stopped after 20–30 min with 50 μL/well of H_2_SO_4_ 2N solution. The absorbance was measured with a Multiskan spectrophotometer at a wavelength of 450 nm (Labsystems, Basingtoke Hants, UK). Three strong positive samples, previously tested by the MZ-iELISA were included in each plate as reference controls and used as duplicates. Additionally, three negative sera reference from a free-tick region were also included as duplicates in each plate. 

In all cases, the absorbance (A) of each serum was expressed as positivity percentage (% P) of th mean value of the positive controls according to the following formula = [(A_450nm_ of serum sample × 100)/Average of A_450nm_ of positive control sera].

### 2.5. Data Analysis

The cutoff point for rMABbo-iELISA was established with the ROC curve (95% confidence interval) using MedCalc Statistical Software version 15.4 (MedCalc Software bvba, Ostend, Belgium; https://www.medcalc.org; 2015). This method allows the estimation of the diagnostic specificity and sensitivity of an established diagnostic method. A frequency distribution graph was also plotted with this program. Concordance between the current ELISA based on *B. bovis* merozoite and rMABbo-iELISA was estimated by Cohen’s kappa value, *k*, as previously described [14].

## 3. Results

**Figure 1:**
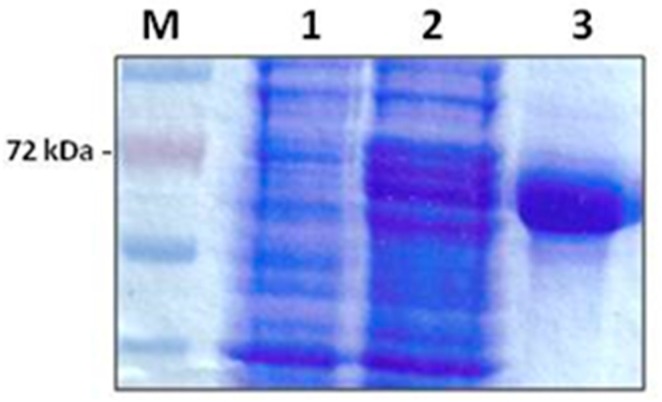
A 12% SDS-PAGE analysis showing the expression and purification of recombinant *B. bovis* multi-antigen, rMABbo. M: Molecular weight marker (Promega). Lane 1: uninduced control; lane 2: level of expression 4 h post-induction with 0, 2% Arabinose; lane 3: purified rMABbo after elution with 250 mM Imidazole.

Purification of the *E. coli* recombinant multi-antigen by Ni-agarose chromatography gave a high purification yield. The purified protein was obtained as a clear band corresponding to the expected molecular weight of 72 kDa in a concentration of 1.2 mg/mL (Figure 1).

**Figure 2:**
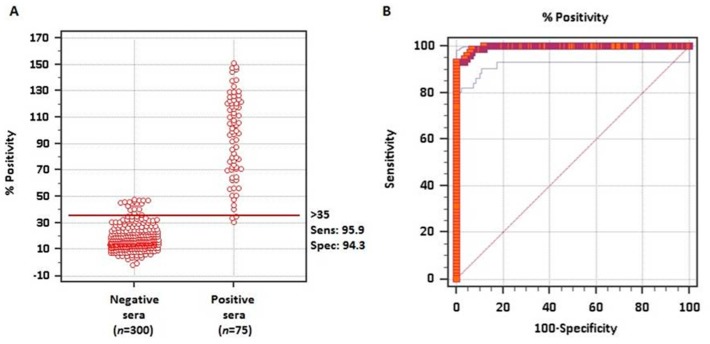
**ROC analysis for the rMABbo-iELISA**. (**A**) Interactive dot plot of the frequency distribution based on 300 known negative sera from tick-free regions of Argentina and 75 known positive sera from experimentally *B. bovis* infected bovines with the attenuated R1A strain. The horizontal line shows the cutoff value of ≥35%, providing a sensitivity of 95.9% and a specificity of 94.3% (**B**). Sensitivity and 100—Specificity values are plotted on the *y*-axis and *x*-axis, respectively. Red thick line indicates the fitted ROC curve and thin blue lines indicate the 95% confidence interval of the fitted ROC curve.

To evaluate whether the rMABbo can be a suitable antigen for the diagnosis of a *B. bovis* infection, the purified protein was initially tested using samples of known serological status. As shown in Figure 2A, using 300 negative sera from tick-free areas and 75 samples from *B. bovis* experimentally infected bovines, the ROC analysis showed an area under the curve, AUC, of 0.995 which means an excellent capability to discriminate truly infected from truly uninfected animals.

The frequency distribution plot of both negative and positive known sera is shown in Figure 2B. With the ROC analysis and establishing a priori a sensitivity of 95.9% and a specificity of 94.3%, a cutoff value of ≥35% was established. With these values only 2 false-negative and 18 false-positive test results were obtained out of the 375 samples analyzed.

In a further step, we evaluated the concordance between rMABbo-iELISA and the MZ-iELISA using a panel of an additional set of 263 bovine sera collected from tick and tick free areas of Argentina. The level of agreement was determined using the Cohen’s *k* value (Table 1). The cross-tabulation between these two tests showed a good level of agreement, resulting in *k* = 0.76 (0.6 < *k* ≤ 0.8; good agreement). Thus, a total of 232 out of 263 sera (85.5%) tested either positive (*n* = 140) or negative (*n* = 92) by both iELISAs, while only 31 samples (11.8%) were discrepant. Twenty-nine of these samples (11%) were only positive for MZ-iELISA whilst only two samples (0.8%) resulted positive for the rMABbo-iELISA being negative for the MZ-iELISA (Table 1).

With the cutoff value of ≥35%, the overall data obtained with this new rMABbo-iELISA showed a high seroprevalence in both Corrientes and Misiones provinces where *R. microplus* is present (Table 2). In Corrientes province, the seroprevalence reached values above 80%, whereas in Misiones the values were close to 70%.

Regarding species specificity, the rMABbo-iELISA was tested with samples originated from regions where the tick *R. microplus* is present and may also transmit other hemoparasites to bovines (i.e., *B. bigemina* and *Anaplasma marginale*).

All the *A. marginale* positive sera (*n* = 5) scored below the cutoff established value and were considered negative. However, of a total of 47 samples serologically positive by the standard iELISA to *B. bigemina*, 33 showed cross-reactivity with the rMABbo protein with a variable percentage of positivity above the cutoff value (between 40% and 80% of positivity).

## 4. Discussion and Conclusions

The serodiagnosis of bovine babesiosis is one of the approaches to control and prevent the dissemination of the disease. At present, there are no commercial ELISA kits for diagnosis of bovine babesiosis caused by either *B. bovis* or *B. bigemina*. In Argentina, the only validated iELISA employs a crude merozoite lysate as the detection antigen. Even though these MZ-iELISA show optimum values of specificity and sensitivity the production of native parasite antigen is difficult, time-consuming and involves maintenance of merozoites in donor bovine red blood cells.

In this work, we have developed an iELISA based on a chimeric polyprotein comprising the immunodominant regions of three *B. bovis* antigens, optimizing the display of multiple B cell epitopes in only one protein. In our previous work, we had observed that all samples of a small group of sera from *B. bovis* naturally-infected bovines recognized the rMABbo in western blots, whereas sera from non-infected cattle did not react [10]. These results led us to move a step further and assay this protein as a detection antigen in an iELISA format.

A common problem observed in diagnosis with indirect ELISAs based on recombinant proteins expressed in prokaryotic systems is the contamination of bacterial proteins with the recombinant antigen, to which bovine serum reacts strongly affecting the interpretation of the results [15]. To overcome this possible drawback we have added an additional blocking mixture of a bacterial lysate to avoid unspecific binding [13]. In our hands, the rMABbo-iELISA showed a low background of unspecific signal and only a few number of truly negative sera resulted as false positive (18 out of 300 samples).

ROC analysis was applied to the rMABbo-iELISA to assess the cutoff value using a batch of samples of known serological status. The cutoff value of 35% obtained in our study is similar to the value obtained by other studies using iELISA based on recombinant antigens and bovine sera [16,17]. Sensitivity and specificity of the rMABbo-iELISA was optimal and very similar to the values reported for the MZ-iELISA [15].

Finally, the concordance of rMABbo-iELISA with the MZ-iELISA in a blind trial test was performed using a panel of an additional set of 263 bovine sera collected in the field from enzootic and non-enzootic areas of Argentina. Although the Cohen’s kappa value between both iELISAs showed a good level of agreement, for a definitive validation, a larger number of samples should be tested to achieve more conclusive results.

Regarding species-specificity, the rMABbo-ELISA showed cross-reactivity with sera from *B. bigemina*—infected cattle. Since the rMABbo includes an *N*-terminal fragment of RAP-1 (amino acids 22 to 236) with 45% of amino acid identity to *B. bigemina* RAP-1, this cross-reaction could be explained by common B cell epitopes between both *Babesia* species. Similar results were obtained by Suarez et al. (1991) when using RAP-1 from *B. bovis* and sera from *B. bigemina*-infected animals [18].

There is also a high amino acid sequence identity (90%) between HSP20 proteins of both *Babesia* species [19], which could also account for the cross-reaction observed in the heterologous sera tested.

This lack of species-specificity due to sequence conservation among antigens chosen for diagnostic purposes is not unusual when recombinant proteins of other parasitic protozoa were used for diagnosis of different species of the same genus. Previous studies of an iELISA for diagnosis of bovine trypanosomosis, showed different level of cross-reactivity when recombinant antigens were used [20]. In addition, a recombinant tandem repeat antigen used in an iELISA for detecting antibodies against surra in water buffaloes, showed cross-reactivity between *Trypanosoma theileri* and *T. evansi* [13]. Another iELISA based on a recombinant antigen against *Theileria* spp. showed cross-reactivity between *T. uilenbergi* and *T. Luwenshuni* in small ruminants [16]. Additionally, in a recent report of an indirect ELISA for the detection of bovine *Theileria* based on three immunodominant proteins, the authors reported a strong cross-reactivity showed in cattle infected with *T. annulata*, *T. orientalis* and *T. sinensis* [17]. Overall, these findings represent one of the major challenges for the selection of immunodominant antigens for diagnostic purposes.

Even though this lack of species-specificity of the rMABbo-iELISA would mean a limitation for the species-specific diagnosis, it must be reminded that detection of either *Babesia* species would require the same intervention at the herd level since there is no difference in the chemotherapeutic treatment of ill animals or management of the tick vector [4]. In this context, our iELISA could be a useful tool for studying the epidemiological status of cattle herds at a regional scale in order to make a prompt decision such as immunization, animal re-location or application of acaricides. In conclusion, the rMABbo-iELISA appears well suited to perform epidemiological surveys like our study in two different regions of Argentina where no previous infection rates are recorded. This would give an overview of the prevalence of the disease and to assess the economic impact in areas where infection with *Babesia* spp. is enzootic.

## 5. Patent

José Manuel Jaramillo Ortiz and Silvina Elizabeth Wilkowsky are named inventors on patent application “Chimeric polypeptide, vaccine against babesiosis, immunization methods, detection methods and kit” WO 2016209859 A1.

## Figures and Tables

**Table 1:** **rMABbo-iELISA and MZ-iELISA blind test on bovine sera from Argentina**. A total of 263 samples were used, 182 of them are originated from endemic areas and 81 from tick-free areas where the vector is not widespread.

	MZ-iELISA		
rMABbo-iELISA	+	−	Total
+	140	2	142
−	29	92	121
Total	169	94	263

**Table 2:** rMABbo-iELISA serological results of serum samples originated from areas where *B. bovis* is endemic.

Origin of the Sera	Positive	Negative	Total	% Prevalence
Misiones	46	22	68	67
Corrientes	98	17	115	85
